# Viral Diversity and Diversification of Major Non-Structural Genes *vif, vpr, vpu, tat* exon 1 and *rev* exon 1 during Primary HIV-1 Subtype C Infection

**DOI:** 10.1371/journal.pone.0035491

**Published:** 2012-05-09

**Authors:** Raabya Rossenkhan, Vladimir Novitsky, Theresa K. Sebunya, Rosemary Musonda, Berhanu A. Gashe, M. Essex

**Affiliations:** 1 Department of Biological Sciences, University of Botswana, Gaborone, Botswana; 2 Botswana–Harvard AIDS Institute, Gaborone, Botswana; 3 Department of Immunology and Infectious Diseases, Harvard School of Public Health, Boston, Massachusetts, United States of America; Massachusetts General Hospital, United States of America

## Abstract

To assess the level of intra-patient diversity and evolution of HIV-1C non-structural genes in primary infection, viral quasispecies obtained by single genome amplification (SGA) at multiple sampling timepoints up to 500 days post-seroconversion (p/s) were analyzed. The mean intra-patient diversity was 0.11% (95% CI; 0.02 to 0.20) for *vif*, 0.23% (95% CI; 0.08 to 0.38) for *vpr*, 0.35% (95% CI; −0.05 to 0.75) for *vpu*, 0.18% (95% CI; 0.01 to 0.35) for *tat* exon 1 and 0.30% (95% CI; 0.02 to 0.58) for *rev* exon 1 during the time period 0 to 90 days p/s. The intra-patient diversity increased gradually in all non-structural genes over the first year of HIV-1 infection, which was evident from the *vif* mean intra-patient diversity of 0.46% (95% CI; 0.28 to 0.64), *vpr* 0.44% (95% CI; 0.24 to 0.64), *vpu* 0.84% (95% CI; 0.55 to 1.13), *tat* exon 1 0.35% (95% CI; 0.14 to 0.56 ) and *rev* exon 1 0.42% (95% CI; 0.18 to 0.66) during the time period of 181 to 500 days p/s. There was a statistically significant increase in viral diversity for *vif* (p = 0.013) and *vpu* (p = 0.002). No associations between levels of viral diversity within the non-structural genes and HIV-1 RNA load during primary infection were found. The study details the dynamics of the non-structural viral genes during the early stages of HIV-1C infection.

## Introduction

The majority of HIV infections in the world, specifically those in sub-Saharan Africa, are attributable to subtype C cementing its status as a major public health concern. Insights into the primary phase of HIV-1C infection are important to better understand disease pathogenesis and early mechanisms of virus-host interactions affecting disease progression. A recent study showed that mutational patterns during the first 5 weeks of infection in a subtype B cohort indicated rapid viral population growth and included a slight decrease in viral genetic diversity over the first 20 to 40 days [1]. Numerous studies have linked viral diversity in HIV-1 structural genes with disease progression [2]–[8]. However, little is known about the diversity and evolution of non-structural genes, which are crucial for virus replication particularly during the early stages of the virus life cycle.

To our best knowledge no published reports to date have pertained to diversity and/or divergence of viral non-structural genes during primary HIV-1 subtype C infection. Salazar-Gonzales et al. showed that the transmitted/founder full length sequences from 12 subjects (9 infected with HIV subtype B and 3 subtype C) contained intact non-structural genes [9]. Michael et al. reported defective accessory genes (HXB2 sequence positions 4,961 to 6,346) in longitudinal HIV-1 subtype B infection [10]. A study on evolution of HIV-1 subtype B Tat and Rev demonstrated that viral mutations were identified within the predicted CTL epitopes suggesting that CTL-mediated selection plays an important role in viral escape from immune pressure and early viral evolution [11]. A longitudinal study on twins infected with HIV-1 subtype B perinatally showed discordant disease progression rates with a dramatic increase in *tat* gene sequence diversity in the sicker child over time [12].

Associations between long term non-progression and viral mutations (including deletions) in HIV-1 non-structural genes have been reported previously [13]–[19]. Yedavalli et al. found that HIV-1 Vif and Vpr were less conserved in their functional domains in a cohort of HIV-1 non-transmitting mothers [20].

A systematic approach to assess the evolution of HIV-1 non-structural genes during primary infection is critical to better understand the role these genes play in HIV-1 pathogenesis and their potential association with early disease progression. This seems particularly important for HIV-1C where a significant fraction of early infections have shown extended periods of high viral RNA load and rapid progression to loss of CD4 lymphocyte numbers and disease progression [21], [22]. This knowledge could inform HIV vaccine design as non-structural viral genes are potentially attractive vaccine candidates. The increased levels of viral replication in HIV-infected individuals rapidly progressing to AIDS can result in greater virus diversity. Although HIV-1 non-structural genes are thought to be relatively conserved, the extent of conservation has been understudied. In this study we assess viral diversity and evolution of five major non-structural HIV-1 genes *vif*, *vpr*, *vpu*, *tat* exon 1 and *rev* exon 1 during the primary phase of HIV-1 subtype C infection up to 500 days post-seroconversion (p/s). We addressed the following questions: (1) What are the phylogenetic relationships of HIV-1 nonstructural genes during primary HIV-1 infection? (2) What is the level of intra-patient diversity during primary infection? (3) How does viral diversity in non-structural HIV-1 genes change over time? and (4) Is HIV-1 RNA load in plasma associated with diversity of HIV-1 non-structural genes during primary HIV-1 infection?

## Methods

### Ethics Statement

This study was conducted according to the principles expressed in the Declaration of Helsinki, and was approved by the Human Research Development Committee of the Botswana Ministry of Health and by the Office of Human Research Administration at the Harvard School of Public Health. All study participants provided written informed consent for the collection of samples and subsequent analysis.

### Study Subjects

Study subjects were enrolled in the primary HIV-1C infection cohort in Botswana and followed-up during 2004–2010 [23]. A subset of 20 adults (eight acutely infected individuals and 12 randomly selected seroconverters) included 5 males and 15 females (Table S1). Age of subjects at enrollment ranged from 20 to 53 years. Acutely infected subjects (patient code A through H) were identified before seroconversion within Fiebig stage II [24] by a negative HIV-1 serology combined with a positive HIV-1 RT-PCR test. Twelve seroconverters (two-digit patient code OC to QR) were identified within the early stage of HIV-1 infection and included 3 subjects within Fiebig stage IV, 4 subjects within Fiebig stage V, 2 subjects on the edge of Fiebig stage V and VI, and 3 subjects in Fiebig stage VI. The time of seroconversion (time 0) was estimated as the midpoint between the last seronegative test and the first seropositive test (approximately one week in most cases) for the acutely infected subjects and by mid-point of the corresponding Fiebig stage for the recently infected subjects [23]. Written informed consent was obtained from all study participants; ethical approval for this research was obtained from the Human Research Development Committee of the Botswana Ministry of Health and OHRA at Harvard School of Public Health.

### Viral RNA Extraction and cDNA Synthesis

Viral RNA was isolated from plasma by QIAamp viral RNA Mini kit (Qiagen, Valencia, CA) according to the manufacturer’s instructions. For viral loads >35,000 copies/ml viral RNA was isolated from 140 µl of plasma, for samples with viral loads <35,000 copies per ml, a volume of plasma containing 5,000 viral RNA copies was spun down at 24,000×g at 4 degrees for 1 hour, the supernatant was removed, the pellet was re-suspended in 140 µl of supernatant for RNA extraction. For samples with viral loads <5,000 copies per ml, the entire aliquot (1.5 mL) was spun down and the pellet was re-suspended in 140 µl of supernatant followed by RNA extraction. Viral RNA was recovered from spin columns in an elution volume of 60 µl. Reverse transcription to single-stranded cDNA was performed using SuperScript III (Invitrogen, Carlsbad, CA) according to manufacturer’s instructions and primer OFM19 (5′ –GCA CTC AAG GCA AGC TTT GAG GCT TA - 3′; HXB2 coordinates 9632 to 9604).

### Single Genome Amplification (SGA) and Sequencing

The single genome amplification was based on the method of limiting dilutions [25], and was used with minor modifications. A median (IQR) of 2.5 (2 to 4) timepoints were sequenced for each patient, and a median of 11.5 (10.4 to 13.5) sequences were generated per patient. The median (IQR) time range of sampling post-seroconversion was 174 (55–349) days. Briefly, the cDNA produced was diluted in 96 well plates with the aim to yield <30% positive reactions of PCR-amplified product. The targeted region spanned from HXB2 nt position 5,041 to 6,310, and included HIV-1 sequence encoding overlapping non-structural viral genes *vif* (nt positions 5,041 to 5,619; HXB2 numbering), *vpr* (nt positions 5,559 to 5,850), *tat* exon1 (nt positions 5,831 to 6,045), *rev* exon 1 (nt positions 5,970 to 6,045) and *vpu* (nt positions 6,062 to 6,310). The diluted cDNA corresponding to about 30% of positive PCR was used as a template for two rounds of nested PCR. The first round PCR was conducted using primers Vif1bw- 5′ – GGG TTT ATT ACA GAG ACA GCA GAG- 3′ (HXB2 coordinates 4900 to 4923) and OFM19, and second-round PCR primers Fvif- 5′ - AGA CCC TAT TTG GAA AGG ACC AGC - 3′ (HXB2 coordinates 4922 to 4945) and Rvpu- 5′-CTT CTT TCC ACA CAG GTA CCC CAT- 3′ (HXB2 coordinates 6366 to 6343). The amplicons were sequenced on both strands. Sequencing primers included Rvpu, Fvif, 5198L- 5′ - TCC AGG GCT CTA GGT TAG - 3′, 4679L- 5′ - GCC CAG GGT CTA CTT GTG - 3′; an additional primer 1466L- 5′ - TCA TTG CCA CTG TCT TCT GCT CTT - 3′ was used to increase coverage in cases primer 5198L failed. Amplicons were Exo-SAP purified [26], and sequenced directly using BigDye technology on the ABI 3730 DNA Analyzer. The sequence fragments were assembled and edited using SeqScape v2.6 (Applied Biosystems). The sequences generated were tested using HYPERMUT v 2.0 [27], and all hypermutated sequences were excluded from the analysis. A total of 6 out of 667 (0.88%) sequences were found to be hypermutated.

During the preliminary analysis of viral quasispecies a link was identified between sequences of subjects OI and OK. We ruled out a potential contamination by (1) similar clustering patterns observed in structural genes, *env* and *gag,* of the same subjects (*env*/*gag* unpublished data from same cohort); (2) similar branching topology of viral sequences obtained at multiple timepoints of sampling; (3) nucleic acid isolation, PCR amplification and sequencing were separated by place and time; and (4) proper quality controls were used in each experiment.

### HIV-1 Subtyping

Nucleotide sequences were codon aligned using the MUSCLE algorithm implemented in Mega 5 [28] followed by manual adjustment in Bioedit [29]. For HIV-1 subtyping, three sequences per patient were randomly selected from the pool of generated viral quasispecies. The selection criteria included earliest timepoint available and sequence length with more than 90% coverage of the targeted region (5,041 to 6,310 nt position in HXB2). To determine phylogenetic relationships and clustering patterns of generated viral sequences, the phylogenetic tree reconstruction was performed using Neighbor joining, Maximum likelihood, and Bayesian methods. A standard set of HIV-1 subtype references from LANL was included in the analysis. The sequence CPZ.CM.98.CAM3.AF115393 was used as an outgroup. The online Rega-2 subtyping tool was used in parallel for confirmation [30].

### Intra-patient Diversity Analysis

The intra-patient mean pairwise distances were measured in Mega 5 using the Maximum Composite Likelihood (MCL) model, following nucleotide sequences alignment using the MUSCLE algorithm implemented in Mega 5 [28] and manual adjustment in Bioedit [29]. Mean values were calculated at each sampling timepoint and the averages of these were used in each time “bin” (0–90, 91–180, 181–500 days p/s). The viral diversity for each non-structural gene was computed as a mean value with 95% Confidence Interval (CI). HIV-1 RNA load in plasma was measured routinely at each study visit over the first year of follow-up. The study schedule included weekly visits for the first two months, bi-weekly for the next two months, and monthly for the next eight months for acutely infected individuals, or monthly for individuals identified within Fiebig stage IV-V. After the first year, quarterly study visits were scheduled for all subjects. Details of HIV-1 RNA load measurement have been presented elsewhere [21], [22], [31]. Mean values for the analyzed time intervals, 0–90 days p/s, 91–180 days p/s, and 181–500 days p/s, and for 100–300 days p/s as described previously, were also included [21]. Potential associations between intra-patient viral diversity and HIV-1 RNA load at the three time intervals, 0–90 days p/s, 91–180 days p/s, and 181–500 days p/s, were assessed.

### Statistical Methods

Sigma plot version 11 was used for descriptive statistics to summarize medians, means, and standard deviations. Comparisons between groups were made using t-test and Mann Whitney Rank sum test for continuous and binary outcomes respectively. All reported p-values are 2-sided. Regression analysis was performed using linear regression and the Spearman Rank Test.

### GenBank Accession Numbers

Sequences have been assigned GenBank accession numbers JQ895561–JQ896230.

## Results

### Inferred Phylogeny of Non-structural Viral Sequences

**Figure 1:**
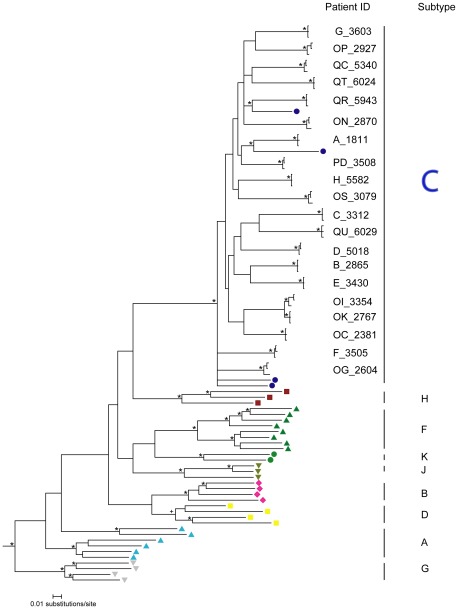
HIV-1 subtyping by analysis of phylogenetic relationships of HIV-1 non-structural genes. The analyzed region of HIV-1 genome corresponded to nucleotide positions 5,041 to 6,310 in HXB2. Three sequences were randomly selected for each study subject (see Methods). A phylogenetic tree was inferred by Mr. Bayes using GTR model. The convergence was reached after 10 M MCMC run. The consensus tree was visualized in Figtree v.1.3.1 [54]. Clade credibility values of >0.95 shown by asterisk, Subtype D cluster showed the support of 0.93 indicated by + symbol. HIV-1 subtype C reference sequences are shown as blue circles. All non-subtype C group M reference sequences are shown at the bottom of the phylogenetic tree. SIV sequence (CPZ.CM98.CAM3.AF115393) was used as an outgroup.

All generated sequences in this study clustered with HIV-1 subtype C reference sequences (Fig. 1). The subject-specific splits were supported by branching topology and clade credibility values of ≥0.99 in the Bayesian analysis. One epidemiologically linked pair, subjects OI and OK, was identified; contamination of samples from subjects OI/OK pair was ruled out.

Good congruence and similar branching topology was observed between the Bayesian (Fig. 1), maximum likelihood (ML; Fig. S1), and neighbor joining (NJ; Fig. S2) tree reconstruction methods. The reliability of the branching topology and clustering patterns was supported by both the approximate likelihood ratio test (aLRT) for ML tree of ≥0.99 and the bootstrap (1,000 replicates) estimates of ≥99% in the NJ analysis. The observed splits support and phylogenetic relationships suggest that the HIV-1 non-structural gene region comprised of *vif*, *vpr*, *vpu*, *tat* exon 1 and *rev* exon 1 can be used reliably for HIV-1 subtyping. The HIV-1 subtyping results were congruent with results obtained using the Rega-2 HIV-1 subtyping tool (data not shown).

**Figure 2:**
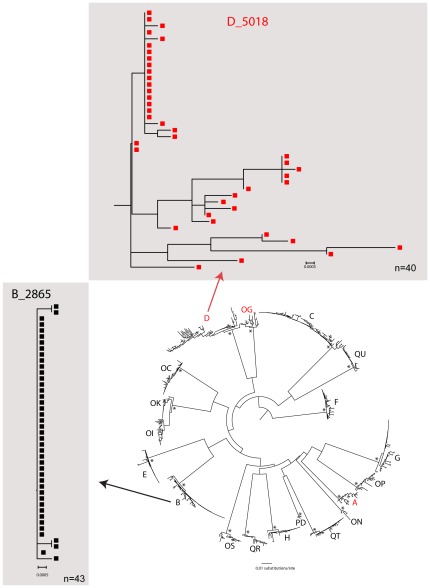
HIV-1 subtype C phylogenetic relationship and diversity of HIV non-structural genes is consistent with the multiplicity of HIV-1 infection determined by analysis of the *env*/*gag* genes. A maximum likelihood phylogenetic tree was reconstructed using Fastree2 (Price *et al*., 2010) using the GTR+G model for nucleotide substitution and visualized in Figtree v.1.1.3 [54]. Alternative likelihood ratio tests [55] >0.95 are shown by an asterisk. Subjects infected with multiple viral variants are colored red. Patient B and D subtrees (individual trees on grey background) show branching topology of earliest sampling (0–90 days p/s) and represent examples of single (subject B) and multiple (subject D) HIV-1 transmission.

To address whether phylogeny of non-structural genes is consistent with multiplicity of HIV-1 transmission, the branching topology of generated viral quasispecies at early timepoints (0–90 days p/s) were analyzed and compared with branching patterns with HIV-1C structural genes, *env* and *gag* [32]. The inferred phylogeny in 3 (15%) subjects included in this study supported transmission of multiple viral variants, and was consistent with our previous findings in *env*/*gag* analysis (Fig. 2). For example, more diversified branching topology was observed in subject D (*env*/*gag*: transmission of multiple viral variants) compared to subject B (*env*/*gag*: transmission of single viral variant).

### Intra-patient Diversity and Diversification of HIV-1C Non-structural Genes During Primary Infection

**Figure 3:**
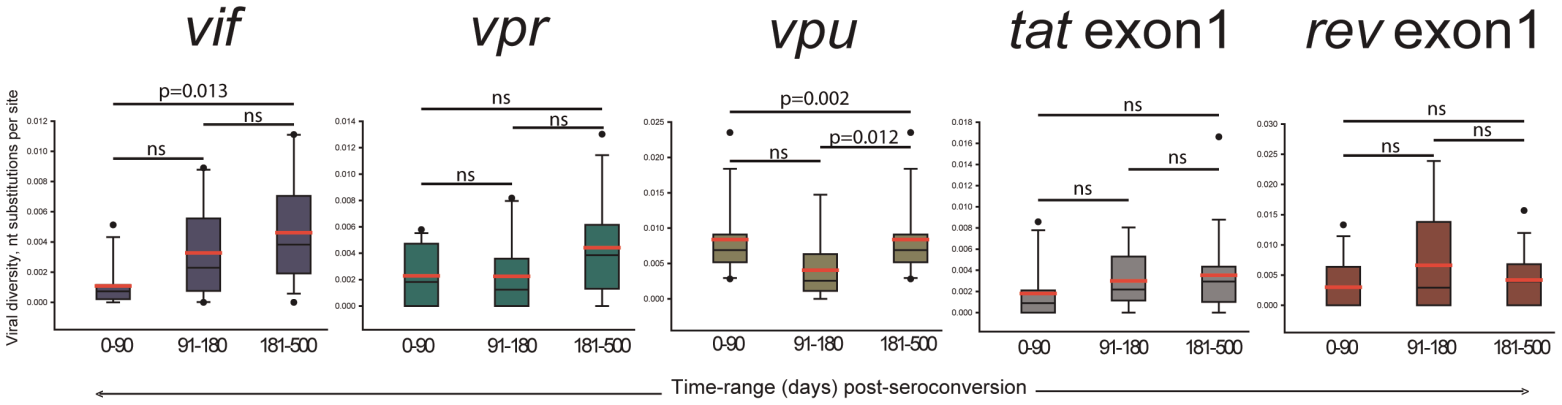
Individual distribution of pairwise distances for each of the non-stuctural genes,*vif* (HXB2 start 5041 to 5619), *vpr* (HXB2 start 5559 to 5850), *vpu* (HXB2 start 6062 to 6310), *tat* exon 1 (HXB2 start 5831 to 6045), and *rev* exon 1(HXB2 start 5970 to 6045).

To assess the level of intra-patient diversity of HIV-1C non-structural genes in primary infection, we used viral quasispecies obtained by SGA at multiple sampling timepoints from seroconversion up to 500 days p/s. The mean intra-patient diversity was 0.11% (95% CI; 0.02 to 0.20) for *vif*, 0.23% (95% CI; 0.08 to 0.38) for *vpr*, 0.35% (95% CI; −0.05 to 0.75) for *vpu*, 0.18% (95% CI; 0.01 to 0.35) for *tat* exon 1 and 0.30% (95% CI; 0.02 to 0.58) for *rev* exon 1 during the time period 0 to 90 days p/s. The intra-patient diversity increased gradually in all non-structural genes (Fig. 3), which was evident from the *vif* mean intra-patient diversity of 0.46% (95% CI; 0.28 to 0.64), *vpr* 0.44% (95% CI; 0.24 to 0.64), *vpu* 0.84% (95% CI; 0.55 to 1.13), *tat* exon 1 0.35% (95% CI; 0.14 to 0.56) and 0.42% (95% CI; 0.18 to 0.66) for *rev* exon 1 during the time period of 181 to 500 days p/s. There was a significant increase in viral diversity calculated using the Mann-Whitney Rank Sum Test for *vif* (p = 0.013) and *vpu* (p = 0.002).

**Figure 4:**
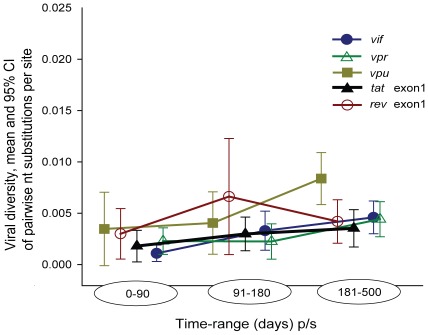
HIV-1C diversity, mean and 95% confidence intervals for non-structural genes *vif*, *vpr*, *vpu*, *tat* exon 1 and *rev* exon 1 over the first 500 days p/s. Viral diversity for each subject was calculated using maximum composite likelihood model [56].

The intra-patient viral diversity of HIV-1C non-structural genes suggested a high level of conservation during primary infection (Fig. 4). A tight range of viral diversity was observed for each non-structural gene analyzed, at the earliest timepoint (0–90 days p/s): *vif* (0%–0.51%), *vpr* (0%–0.58%), *vpu* (0%–1.98%), *tat* exon 1 (0%–0.86%) and *rev* exon 1 (0%–1.33%). At later timepoints, over the first year of HIV1-C infection, an increase was also observed, the range of viral diversity was tight: *vif* (0%–1.11%), *vpr* (0%–1.30%), *vpu* (0.28%–2.35%), *tat* exon 1 (0%–1.66%) and *rev* exon 1 (0%–1.57%). To assess the change in diversity over time, slopes of changes were analyzed. The gradual increase observed by positive slopes of viral diversity (*vif*, 0.40%; *vpr*; 0.25%; *vpu*, 0.61%; *tat* exon1, 0.19%; and *rev* exon1, 0.05%) is consistent with ongoing viral evolution.

### HIV-1 RNA Load and Diversity within HIV-1C Non-structural Genes

The potential association between intra-patient non-structural gene diversity and levels of viral RNA load during primary HIV-1C infection was examined. It was assumed that higher levels of viral replication during the early stages of infection could drive increased viral diversity at later timepoints. The levels of intra-patient diversity within *vpr* and HIV-1C RNA load at 0–90 days p/s showed a weak but statistically significant correlation (r^2^ = 0.587, p = 0.042). The intra-patient diversity within *tat* exon 1 and *rev* exon 1 at later timepoints (181–500 days p/s) was associated with HIV-1 RNA load at the baseline timepoint, r^2^ = 0.505, p = 0.044; and r^2^ = 0.577, p = 0.019, respectively. There was association between *tat* exon 1 diversity and HIV-1 RNA load at 181–500 days p/s, r^2^ = 0.516, p = 0.039. Thus we found only sporadic associations between levels of HIV-1 RNA load and intra-patient viral diversity within non-structural genes. This can be attributed to the relatively short region of analyzed viral genes, fewer timepoints analyzed, uneven number of generated quasispecies, or missing the region encoding the second exons for Tat and Rev.

The potential difference in intra-patient diversity within HIV-1C non-structural genes between extended high viremics [21] and other patients during primary HIV-1C infection were also examined. Levels of intra-patient diversity between these two groups of subjects were compared at three time intervals, 0–90, 91–180, and 181–500 days p/s. We hypothesized a higher level of viral diversity within the HIV-1C non-structural genes in extended high viremics; however, no significant differences between groups were found (Fig. S3).

## Discussion

This study performed a comprehensive analysis of the molecular evolution of HIV-1C non-structural genes during primary infection from seroconversion up to 500 days p/s. To our best knowledge, this is the first report on the extent and dynamics of viral diversity within HIV-1C non-structural genes during primary HIV-1C infection. Also recent studies suggest that HIV-1C infections may show unusually high HIV RNA levels for prolonged periods following initial infection.

A limited number of studies have addressed viral quasispecies in HIV-1C infection [33]–[35], although none has focused on the evolution of HIV-1C non-structural genes. While the diversity of HIV-1C non-structural genes has been addressed in previous cross-sectional studies [36]–[38], these studies were not powered to evaluate the extent of viral diversification over time. The use of the SGA method in this study allowed the assessment of longitudinal diversity and diversification of non-structural genes during primary HIV-1C infection.

Intra-patient nucleotide diversity increased over time in all non-structural genes studied, which is consistent with previously published data on *env* gp120 in primary HIV-1B [8] and HIV-1C [23] infection. The tight range observed in *vif* (0%–0.51%), *vpr* (0%–0.58%), *vpu* (0–1.98%), *tat*1 (0%–0.86%) and *rev*1 (0%–1.33%) at the earliest timepoint which increased slightly up to 500 days p/s *vif* (0%–1.11%),*vpr* (0%–1.30%),*vpu* (0.28%–2.35%), *tat* exon1 (0%–1.66%) and *rev* exon1 (0%–1.57%) highlights the high level of viral conservation in HIV-1 non-structural genes. Viral diversification in non-structural genes is lower than in structural HIV-1 genes, which is evident from comparison of slopes with the slope in HIV-1 B*env* reported at 1% per year [8]. Previous studies have assessed whether viral mutations occur within CTL epitopes and/or flanking regions; however, most previous studies analyzed primarily structural viral genes, and evolution of CTL epitopes within accessory genes remains understudied [1], [39]–[44]. Therefore, it remains unclear to what extent selection pressure drives evolution of the HIV-1 non-structural genes. Although data from cross-sectional HIV-1C studies have shown a high level of conservation in major non-structural genes [36]–[38].The lower level of non-structural genes diversity during the primary infection is consistent with known viral homogeneity during this period [23], [45]–[48].

We found that the HIV-1 genome region encoding non-structural viral genes can be used reliably for HIV-1 subtyping, which is consistent with previous studies [36], [49]–[51]. The sporadic associations between non-structural HIV-1C gene diversity and viral RNA load in plasma is consistent with previous studies [4], [52], [53]. The viral diversity in HIV-1C non-structural genes was consistent with multiplicity of viral transmission and results in the *env*/*gag* analysis [32]. The analyzed timepoints allowed us to detect transmission of more than a single viral variant. However, we were not able to deduce the number of transmitted viral variants due to potential recombinations between transmitted variants, if samples were collected later than Fiebig stage IV.

The relatively small sample size and different number of generated viral quasispecies per timepoint and/or per subject are the major limitations in this study. Ideally, it would have been preferable to obtain at least 20 sequences for each patient at each timepoint. However, this task was challenging and difficult to achieve due to inter-subject heterogeneity and viral RNA as a template for amplification. An additional limitation of the study is the overlapping nature of the proteins in the region amplified, making it difficult to assign specific mutational events to specific proteins. Also, the second exon of tat and rev were not studied.

The study provides insights into the dynamics of the non-structural HIV-1C genes during the early stages of infection. The results of our study highlight differential diversity across HIV-1 genes and slower diversification of viral accessory genes over time. Apparently, the most likely reason is different selection pressure imposed by host immune response to the encoded viral gene products which may result in different evolutionary rates. Our results suggest (1) a high level of conservation of viral non-structural genes during primary HIV-1C infection; (2) a gradual increase in viral diversity of these genes over the first 500 days p/s; and (3) no associations between levels of viral diversity within the non-structural genes and HIV-1 RNA load during primary infection, which might be due to a relatively small sample size. To conclusively evaluate potential associations, larger and more in-depth studies might be warranted. This could yield valuable information to aid vaccine development, due to the increasing interest of non-structural genes as targets in vaccine design.

## Supporting Information

Figure S1 HIV-1 subtyping. Phylogenetic relationship between HIV-1 non-structural genes. A phylogenetic tree was constructed using PhyML [49] using the GTR+I+G model for nucleotide substitution and visualized in Mega 5 [27]. Three sequences were used for each patient Subjects’ branches are labeled on the right with patient codes. aLRT >0.99 shown by asterisk. HIV-1 subtype C reference sequences are shown in blue, and all other HIV-1 group M (non-C) reference sequences are labeled at the bottom of the figure. SIV sequence (CPZ CM98.CAM3.AF115393) was used to root the tree.
(TIF)

Figure S2 HIV-1 subtyping. Phylogenetic relationship between HIV-1 non-structural genes. A phylogenetic tree was constructed from nucleotide alignments using Neighbor Joining (NJ) method. The evolutionary distances were computed using the Kimura 2-parameter method. The reliability of the branching topology was estimated from 1000 bootstrap replicates. Patient identifiers are shown to the right of the tree. Bootstrap values >99% are indicated by asterisks. SIV sequence (CPZ CM98 CAM3 AF115393) was used to root the tree.
(TIF)

Figure S3 Viremics Analysis; Kimura 2-parameter overall mean pairwise diversity of HIV-1C non-structural genes *vif*, *vpr*, *vpu*, *tat* exon1 and *rev* exon1 comparing two groups (high viremics – individuals with mean HIV-1 RNA load >100,000 copies/ml during the period 100–300 days p/s, and other subjects).
(TIF)

Table S1 Subject demographics, time points of sampling, HIV-1 RNA load, CD4 count, and Fiebig stage at enrollment.
(DOCX)
