# Supplementary figures and images for: Viral Diversity and Diversification of Major Non-Structural Genes vif, vpr, vpu, tat exon 1 and rev exon 1 during Primary HIV-1 Subtype C Infection
Source: PLoS One. 2012 May 9;7(5):e35491. doi: 10.1371/journal.pone.0035491 (PMC3348911; doi:10.1371/journal.pone.0035491)

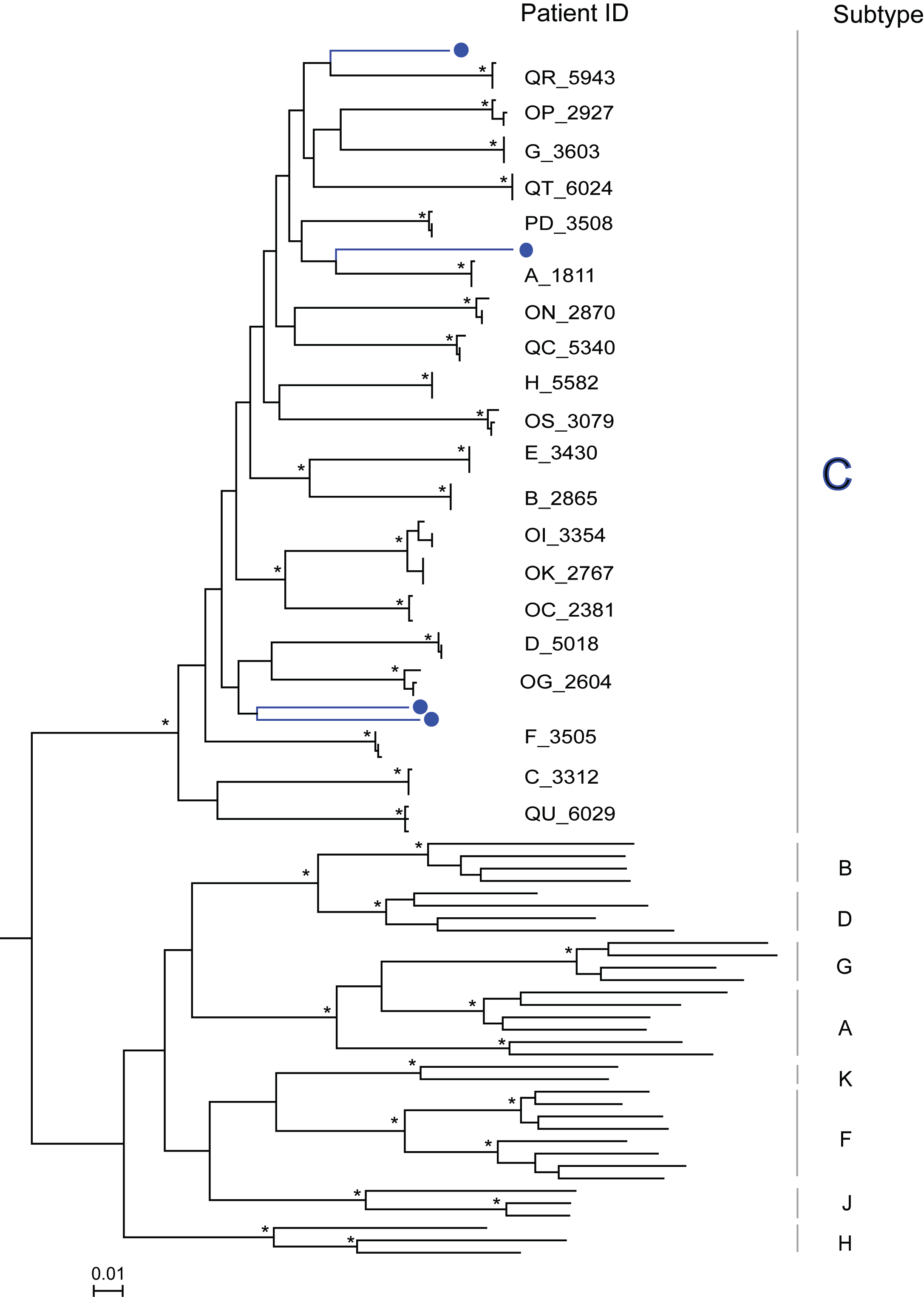

Supplement: Figure S1 — HIV-1 subtyping. Phylogenetic relationship between HIV-1 non-structural genes. A phylogenetic tree was constructed using PhyML [49] using the GTR+I+G model for nucleotide substitution and visualized in Mega 5 [27]. Three sequences were used for each patient Subjects’ branches are labeled on the right with patient codes. aLRT >0.99 shown by asterisk. HIV-1 subtype C reference sequences are shown in blue, and all other HIV-1 group M (non-C) reference sequences are labeled at the bottom of the figure. SIV sequence (CPZ CM98.CAM3.AF115393) was used to root the tree. (TIF) [file pone.0035491.s001.tif]

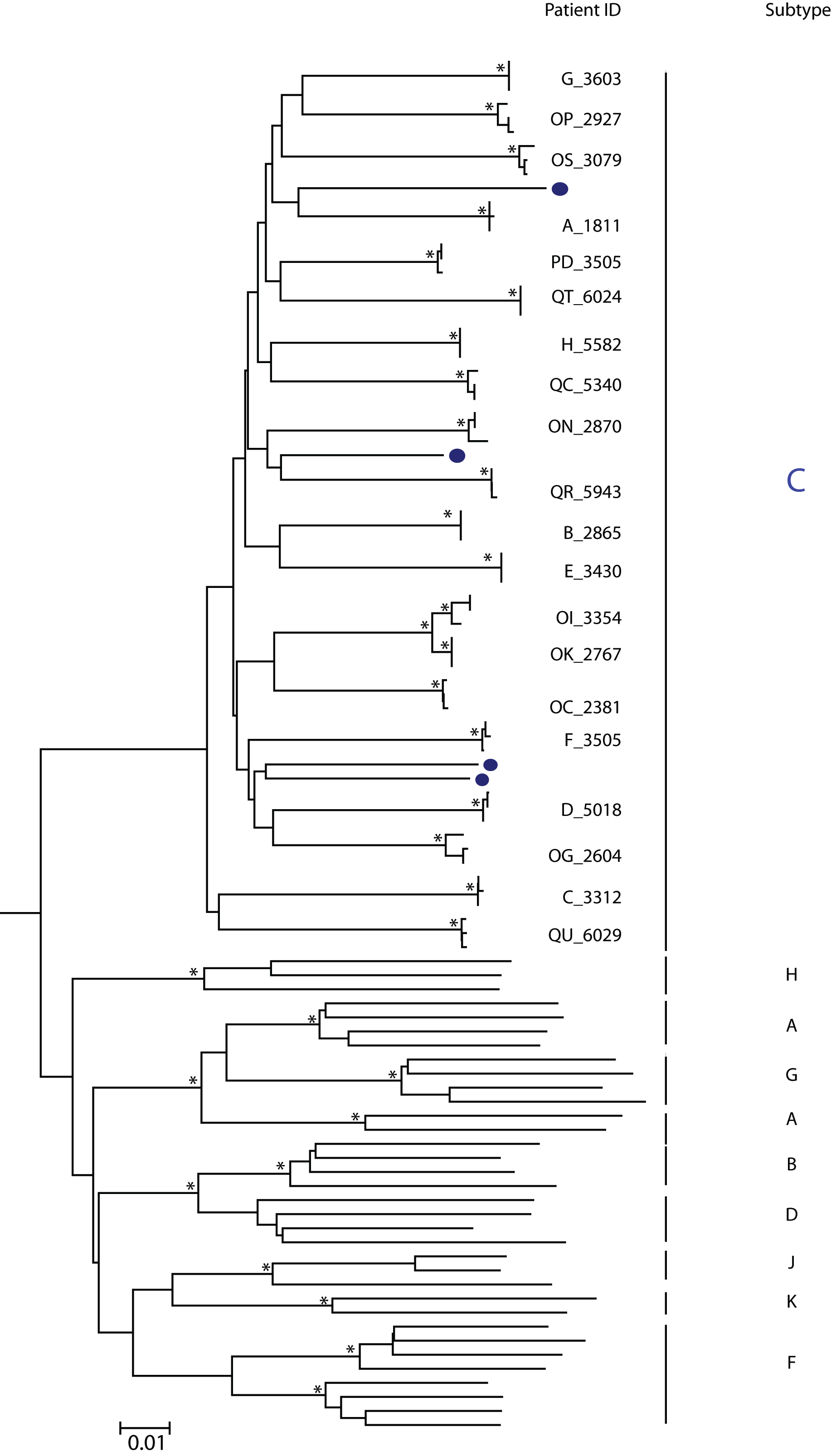

Supplement: Figure S2 — HIV-1 subtyping. Phylogenetic relationship between HIV-1 non-structural genes. A phylogenetic tree was constructed from nucleotide alignments using Neighbor Joining (NJ) method. The evolutionary distances were computed using the Kimura 2-parameter method. The reliability of the branching topology was estimated from 1000 bootstrap replicates. Patient identifiers are shown to the right of the tree. Bootstrap values >99% are indicated by asterisks. SIV sequence (CPZ CM98 CAM3 AF115393) was used to root the tree. (TIF) [file pone.0035491.s002.tif]

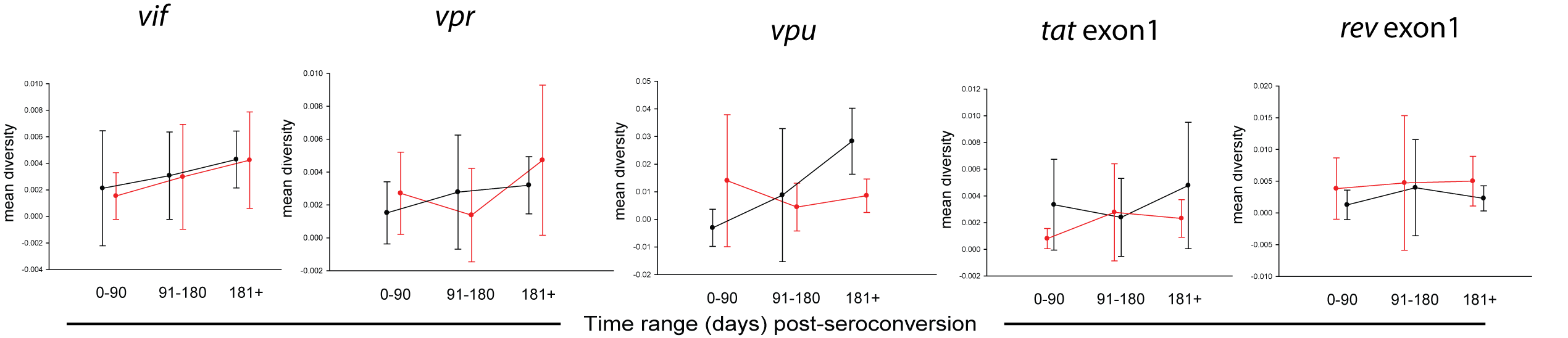

Supplement: Figure S3 — Viremics Analysis; Kimura 2-parameter overall mean pairwise diversity of HIV-1C non-structural genes vif, vpr, vpu, tat exon1 and rev exon1 comparing two groups (high viremics – individuals with mean HIV-1 RNA load >100,000 copies/ml during the period 100–300 days p/s, and other subjects). (TIF) [file pone.0035491.s003.tif]
