# Supplementary material for: Viral Diversity and Diversification of Major Non-Structural Genes vif, vpr, vpu, tat exon 1 and rev exon 1 during Primary HIV-1 Subtype C Infection
Source: PLoS One. 2012 May 9;7(5):e35491. doi: 10.1371/journal.pone.0035491 (PMC3348911; doi:10.1371/journal.pone.0035491)
Supplement: Table S1 — Subject demographics, time points of sampling, HIV-1 RNA load, CD4 count, and Fiebig stage at enrollment. (DOCX) [file pone.0035491.s004.docx]

Table S1**:**  Subject demographics, timing and depth of sequence analysis.

| Patient ID | Age | Gender | HIV-1 RNA viral load log10 copies/ml at enrollment | CD4 count at enrollment | Fiebig stage at enrollment | Total number of sequences generated | Time of sampling estimated days p/s (HIV-1 RNA sequences, n) | | | | | | | | | | | | Average number of sequences | Number of samplings |
| --- | --- | --- | --- | --- | --- | --- | --- | --- | --- | --- | --- | --- | --- | --- | --- | --- | --- | --- | --- | --- |
| A_1811 | 31 | F | 6.09 | 172 | II | 19 | 6 | (4) | 83 | (1) | 147 | (8) | 181 | (6) |  |  |  |  | 4.8 | 4.0 |
| B_2865 | 20 | F | 5.74 | 373 | II | 71 | 10 | (39) | 39 | (4) | 103 | (2) | 199 | (24) | 223 | (2) |  |  | 14.2 | 5.0 |
| C_3312 | 32 | F | 6.56 | 202 | II | 92 | 4 | (19) | 109 | (18) | 201 | (46) | 362 | (9) |  |  |  |  | 23.0 | 4.0 |
| D_5018 | 27 | M | 6.32 | 286 | II | 77 | 6 | (34) | 34 | (6) | 103 | (8) | 177 | (14) | 393 | (3) | 483 | (12) | 12.8 | 6.0 |
| OC_2381 | 25 | M | 5.08 | 301 | IV | 41 | 27 | (3) | 208 | (36) | 393 | (2) |  |  |  |  |  |  | 13.7 | 3.0 |
| OG_2604 | 23 | F | 6.47 | 260 | V | 21 | 44 | (15) | 418 | (6) |  |  |  |  |  |  |  |  | 10.5 | 2.0 |
| QU_6029 | 25 | F | 5.42 | 551 | V-VI | 27 | 114 | (12) | 174 | (15) |  |  |  |  |  |  |  |  | 13.5 | 2.0 |
| E_3430 | 35 | F | 5.38 | N/A | II | 33 | -30 | (29) | 150 | (2) | 372 | (2) |  |  |  |  |  |  | 11.0 | 3.0 |
| F_3505 | 53 | F | 5.57 | 426 | II | 49 | 7 | (34) | 352 | (15) |  |  |  |  |  |  |  |  | 24.5 | 2.0 |
| G_3603 | 34 | M | 5.76 | 505 | II | 46 | 4 | (21) | 32 | (1) | 108 | (6) | 227 | (10) | 438 | (8) |  |  | 9.2 | 5.0 |
| H_5582 | 26 | F | 5.18 | 570 | II | 32 | 11 | (8) | 16 | (15) | 35 | (2) | 202 | (2) | 378 | (5) |  |  | 6.4 | 5.0 |
| OI_3354 | 30 | F | 3.79 | 410 | IV | 27 | 117 | (3) | 294 | (23) | 476 | (1) |  |  |  |  |  |  | 13.0 | 3.0 |
| OS_3079 | 42 | M | 3.18 | 258 | V | 20 | 243 | (5) | 411 | (15) |  |  |  |  |  |  |  |  | 10.0 | 2.0 |
| PD_3505 | 25 | F | 4.52 | 1161 | V | 5 | 59 | (5) |  |  |  |  |  |  |  |  |  |  | N/A | 1.0 |
| QR_5943 | 26 | F | 2.6 | 801 | IV | 23 | 90 | (10) | 485 | (13) |  |  |  |  |  |  |  |  | 11.5 | 2.0 |
| QT_6024 | 24 | F | 4.89 | 522 | V | 32 | 137 | (13) | 289 | (8) | 349 | (11) |  |  |  |  |  |  | 10.7 | 3.0 |
| OK_2767 | 26 | M | 4.91 | 251 | VI | 27 | 117 | (26) | 299 | (1) |  |  |  |  |  |  |  |  | 13.5 | 2.0 |
| ON_2870 | 36 | F | 2.6 | 577 | VI | 5 | 488 | (5) |  |  |  |  |  |  |  |  |  |  | N/A | 1.0 |
| OP_2927 | 26 | F | 4.68 | 436 | VI | 22 | 304 | (10) | 486 | (12) |  |  |  |  |  |  |  |  | 11.0 | 2.0 |
| QC_5340 | 22 | F | 3.86 | 658 | V | 3 | 80 | (3) |  |  |  |  |  |  |  |  |  |  | N/A | 1.0 |
